# Unraveling the effect of ambivalence over emotional expression on subjective wellbeing in Chinese college students in romantic relationships: A multiple mediation model

**DOI:** 10.3389/fpsyt.2022.982406

**Published:** 2022-09-08

**Authors:** Yue Wang, Zexin Zheng, Xiaoyu Wang, Ying Li

**Affiliations:** School of Education, Zhengzhou University, Zhengzhou, China

**Keywords:** ambivalence over emotional expression, subjective wellbeing, fear of intimacy, attachment avoidance, COVID-19

## Abstract

The sudden and unpredictable outbreak of the COVID-19 pandemic has severely threatened young adults’ physical and mental health and damaged the quality of relationships. As a critical stage of development, their well-being is more vulnerable to adverse environments which may lead to profound negative long-life mental health status. The current study aimed to investigate the mediation effects of fear of intimacy and attachment avoidance in the association between ambivalence over emotional expression and subjective wellbeing. A sample of 555 Chinese college students who are currently involved in romantic relationships (Mean age = 19.69, SD age = 1.36, 52% females) completed anonymous questionnaires regarding ambivalence over emotional expression, fear of intimacy, attachment avoidance, and subjective wellbeing. The findings revealed that ambivalence over emotional expression can predict negative subjective wellbeing of college students in romantic relationships significantly, and fear of intimacy and attachment avoidance played a sequentially mediating role in the association between ambivalence over emotional expression and subjective wellbeing. Surprisingly, when considering attachment avoidance independently, we found it had an inhibitory action on the link between ambivalence over emotional expression and subjective wellbeing. The present study contributes to a better understanding of how ambivalence over emotional expression can decrease subjective wellbeing, and also has implications for the intervention of students’ subjective wellbeing and closeness during the period of COVID-19.

## Introduction

The global outbreak of Coronavirus disease 2019 (COVID-19) has severely threatened the psychological and physical wellbeing of people all over the world (1). Particularly, the long-term lockdown and isolation measures have negatively affected individuals’ interpersonal security (2). As a critical developmental stage, young adults show high sensitivity to environmental influences (3), and previous studies have indicated that their subjective wellbeing significantly declined during the pandemic (4). Subjective wellbeing (SWB) is defined as an individual’s general appraisal of their lives, which consists of cognitive judgments regarding their overall life satisfaction and emotional reactions including positive and negative emotions, and it reflects whether an individual lives a satisfied and desirable life (5) and is also an important indicator of positive psychological development and mental health. Previous studies have shown that individuals with higher SWB lead longer, healthier lives, together with a lower rate of suicide ideation (6). By contrast, the decline in SWB may lead to a higher incidence of depression, social anxiety, and insecurity (7, 8). Notably, for the youth, subjective wellbeing has been found to be particularly vulnerable to adverse social circumstances (2). For example, youths who fail in maintaining a supportive social environment usually lived in an isolating social environment which could seriously undermine their emotional or psychological wellbeing (9). Meanwhile, prior studies have found people who are unable to regulate their emotions tend to have trouble maintaining a supportive environment (10), and their improper expression of feelings may result in more unfulfilled needs (11). What is worse, based on the model of context-process-outcome (12), negative personal disposition might exacerbate the impact of the environment and lead to more severe psychological problems because people need more emotional support to mitigate distress in difficult times, such as COVID-19 period.

Besides, a decreasing trend could also be seen in college students’ experience of romantic relationships during the COVID-19 period due to the lockdown policy which reduced the amount of time staying together (13), with Vigl and colleagues attributing this to limited time staying together under lockdown policy for non-cohabiting couples (college students). Moreover, under such stressful circumstances, positive emotions and expression of feelings might have a beneficial effect on individuals’ attitudes toward their partners, which are highly correlated with their romantic experience and subjective wellbeing (14, 15). However, college students with poor emotional expressing ability may not only be awash with insufficient emotional expression but also distort their attitudes in relationships, leading to a negative influence on subjective wellbeing.

In a word, poor emotional expression may have a greater negative impact on the subjective wellbeing of students who involve in romantic relationships. Therefore, the present study aimed to investigate the relationship between a particular psychological risk factor called ambivalence over emotional expression and subjective wellbeing among college students in love during the COVID-19 pandemic. Given the serious effect of the pandemic, the present study could provide a more comprehensive insight into the adverse psychological impact of insufficient emotional expression and propound some practical and feasible interventions to mitigate that situation.

### Ambivalence over emotional expression and subjective wellbeing

Ambivalence over emotional expression (AEE) refers to an internal conflict of expressing one’s positive or negative feelings in fear of negative consequences from exhibiting such expression (16). Previous studies have shown that AEE is associated with high levels of psychological distress, and depressive and anxiety symptoms (17). More specifically, King and Emmons (16) stated that individuals with high AEE are likely to overread and overthink other’s reactions in social interaction, and their rumination over potential negative feedback from others turn into a stressor in their minds. Furthermore, given their inability to express their feelings and emotions properly, they could not or hardly take self-disclosure as an effective coping strategy when experiencing negative moments in their lives (18), so that they are unable to use social support as a coping mechanism, which leads to a lack of effective strategies to manage stressful life events. On the other hand, some internalizing symptoms like depression and anxiety, which can trigger emotional problems, are also highly correlated with AEE (17). Therefore, it can be seen that people with high AEE are more likely to have both emotional and cognitive problems, both of which are the core components of subjective wellbeing (5). During the outbreak of the COVID-19 pandemic, college students tend to experience more stressful events in both economic and psychological aspects, such as decreasing family income, low living quality, and insecurity (8, 19), which means people with high AEE may struggle to find emotional support to deal with these stresses, which might cause a prominent decline in subjective wellbeing. As a result, it is important for recent studies to illustrate the underlying association between AEE and SWB in order to put forward related interventions.

Since AEE can reflect normative responses to negative experiences, it may also be influenced by local culture (20). For instance, emotional expression in East Asian cultures is generally considered to be a sign of weakness and lack of self-control in a social environment (21), whereas western cultures encourage self-disclosure (22). Based on the theory of person–culture fit, a match between one attitude/value and the prevailing attitude/value of the cultural environment in which he or she lives is beneficial to individuals’ wellbeing (23). Therefore, in the context of Chinese culture, the relationship between AEE and subjective wellbeing still remains inscrutable, which can be neutral or even positive. So, the present study aimed to investigate whether or not AEE can significantly predict a decline in subjective wellbeing among the population of Chinese college students.

### The mediating role of fear of intimacy

Researchers suggested that AEE was also associated with marital dissatisfaction in couples (24), which provides new insight into how AEE influences the subjective wellbeing of college students through romantic relationship attitudes, as they are at a critical juncture of identity development and relationship exploration (25), and their attitudes toward romantic partners largely affect their life satisfaction in life span (14).

Given that the exploration and development of closeness is an important goal of college students (26), a major obstacle to achieving this goal is fear of intimacy (FOI). Fear of intimacy can be conceptualized as a limited ability or willingness to share personal feelings or emotions with someone who is highly valued (27). Prior work showed that fear of intimacy is usually significantly associated with a host of negative outcomes in relations (28). To be specific, various studies confirmed that fear of intimacy might give rise to various psychological disorders [e.g., anxiety, social phobia, depression, post-traumatic stress disorder, substance use, and hoarding symptoms; (29–31)], which eventually lead to the drop of subjective wellbeing. Although fear of intimacy is mostly limited to particular emotional suppression in romantic relationships, Toh et al. (32) found that people with a high level of FOI generally have decreased social connectedness, which would ultimately lead to a decline in perceived social support and an increase in loneliness in a more general relationship (33), resulting in the lower level of subjective wellbeing. Based on the studies mentioned above, it can be seen that fear of intimacy can contribute to a poorer assessment of individuals’ lives, which may deteriorate people’s subjective wellbeing, both in social and individual aspects.

On the other hand, it has been found that people with high AEE may develop emotional suppression as a strategy to deal with conflicting emotions, and such inhibition strategy has been wildly verified in general interpersonal interaction (18). However, as a primary and unique relationship in adulthood (34), romantic relationships can play a pivotal role in difficult or stressful times (35), so it is unclear whether or not the same suppression strategy resulting from AEE can be applied to romantic relationships. If so, it could be concluded that even when facing their important partners, they still tend to overestimate the possibility of rejection, wrongly believing that their partner would reject their expression and eventually hurt their feelings (16). Besides that reason, researchers also found that conflict over emotional expression might interrupt the processing and experience of emotions (11), which means people with high AEE may find difficulty in detecting emotions due to a lack of emotional awareness. However, whether or not AEE could lead to stronger fear of intimacy still need to be verified, as well as the mediating role of fear of intimacy between AEE and subjective wellbeing.

Taken together, we proposed that AEE positively predicts fear of intimacy, which in turn reduces subjective wellbeing, namely, fear of intimacy mediates the association between AEE and subjective wellbeing.

### The mediating role of attachment avoidance

Another important factor contributing to SWB is insecure attachment. According to the classification by Bartholomew (36), adult insecure attachment types include anxious attachment and avoidant attachment. The latter tends to perform avoidant behaviors in interpersonal relationships and prevent themselves from a close individual (37). Previous studies have reported that attachment avoidance was negatively correlated with subjective wellbeing (38). Indeed, Kalkotan (39) found that college students with attachment avoidance could predict lower life satisfaction, which is the key component of subjective wellbeing. Additionally, there is a negative association between attachment avoidance and relationship quality or satisfaction in cross-sectional studies (40, 41) and longitudinal studies. For example, Fitzpatrick and Lafontaine (42) investigated 199 Canadian heterosexual couples by administering questionnaires for a period of 3 years, finding that attachment avoidance and anxiety predict lower relationship satisfaction, which could eventually lead to a decline in subjective wellbeing.

Additionally, people with high AEE usually experience conflicts and difficulties when longing for expressing negative or even positive emotions and feelings to others, because they are scared of negative feedback from others after expression. Also, it is important to be demonstrative and receive appropriate responses when maintaining a romantic relationship (33). So, such conflicts toward expressing emotions undoubtedly would undermine one’s faith in forming or maintaining romantic relationships and reinforces their sense of incompetence. Feeney (43) asserted that attachment avoidance mainly results from the fear of one’s own incompetence in a romantic relationship. Therefore, it can be assumed that people with high AEE usually feel incompetent in romantic relationships, which eventually tends to develop severe attachment avoidance.

Based on the preceding studies, we assumed that attachment avoidance mediates the association between AEE and subjective wellbeing. Specifically, AEE positively predicts attachment avoidance, which in turn reduces subjective wellbeing.

### The sequential mediation model

The interpersonal model of intimacy (44) highlights the importance of self-disclosure and partners’ responsiveness in romantic relationships. From this perspective, individuals engage in disclosure of self-relevant feelings or emotions and then receive others’ responses, which makes individuals feel understood, validated, or cared for. Such successful interaction may be viewed as a rewarding relationship (45). However, people with a fear of intimacy tend to suppress their emotional expression in romantic relationships, which means there is no satisfying self-disclosure related to emotions or feelings between romantic partners, let alone emotional support from partners. At this point, people tend to perceive such relationships as unrewarding, which eventually might lead to more avoidant behaviors in a relationship (46, 47). In addition, the effort to hide one’s emotions may involve less sensitivity to another person’s pain (48), so people who have great FOI might fail in supporting their romantic partners in the emotional field, which is important when forming an intimate relationship. Consequently, they might find difficulties in maintaining their relationships, which can also contribute to avoidant behaviors (43). Such difficulties may lead to a more severe consequence during the time of COVID-19 when people need to go through psychological and physical hardships. Therefore, it can be assumed that fear of intimacy and attachment avoidance play sequential mediating roles in how ambivalence affects subjective wellbeing.

However, when considering the relationships between fear of intimacy and attachment avoidance, some researchers hold the opposite view. Given attachment process can shape an individual’s beliefs and interactions in interpersonal relationships, thus affecting the individual’s overall evaluation of life (49), previous studies have also found that attachment avoidance could lead to emotional inhibition strategy in relationships (37, 50). More specifically, people with attachment avoidance tend to regard the experience/expression of emotions (both negative and positive) as a vulnerable indicator of interpersonal closeness, and hence they are inclined to suppress them (51). So, we attempted to further investigate the effects of fear of intimacy and attachment avoidance on subjective wellbeing, considering fear of intimacy as a mediator.

### The present study

The current study aimed to test the following aspects: (a) whether AEE could negatively predict subjective wellbeing, (b) whether fear of intimacy mediated the relationship between AEE and subjective wellbeing, (c) whether attachment avoidance mediated the association between AEE and subjective wellbeing, and (d) whether fear of intimacy and attachment avoidance work as the sequential mediation between AEE and subjective wellbeing.

## Materials and methods

### Participants

After being approved by the Ethical Committee for Scientific Research at the authors’ institution (Ethical Committee of Zhengzhou University), the current study was conducted at three universities in Zhengzhou City, Henan Province, China. A convenient sampling method was conducted, and a total of 555 students (268 men, mean age = 19.69 years, SD = 1.36, range from 17 to 23 years) who were involved in romantic relationships currently were able to join this survey. An item was asked to select the qualified participants (“How long have you been in current romantic relationships”), we excluded students who chose “0,” and among the qualified students, 107 (19.3%) chose 1–3 months, 191 (34.4) chose 3 months - 1 year, 174 (31.4%) chose 1–3 years, and 83 (15%) chose more than 3 years. We assigned research assistants to each of the universities to organize the survey. They informed the students about the time and place of the survey and asked the students to start the measurement in their classrooms. After finishing the survey, all participants were provided with payments. The details of participants’ characteristics are shown in Table 1.

**TABLE 1:** Participants’ characteristics (*n* = 555).

	Age	Gender		Grade			
		Male	Female	Freshmen	Sophomore	Junior	Senior
Mean or *n*	19.69	268	287	106	300	148	1
±SD or %	1.36	48.29%	51.71%	19.09%	54.05%	26.67%	0.18%

### Measurement

#### Ambivalence over emotional expression

Ambivalence over emotional expression was measured by the Chinese version of the AEQ scale (16), which consists of 28 items. It measures expressions of positive emotions, negative emotions, and intimacy (e.g., “I want to express my emotions honestly but I am afraid that it may cause me embarrassment or hurt.”). Participants respond to items on a 5-point Likert scale ranging from 1 = never to 5 = frequently, indicating how often they feel what each statement suggests. The overall score was the summation of all items, with a higher score representing people feeling more conflict when they try to express their feelings. The AEQ has been used in Chinese samples with good reliability and validity (52). In the present study, the Cronbach’s alpha coefficient for this scale was 0.906 [95 % CI (0.232, 0.282)].

#### Fear of intimacy

Fear of intimacy was measured by the Fear of Intimacy Scale [FIS; (27)], which is a 35-item scale that reports on the degree to which participants are uncomfortable with or fear intimacy in their relationships [e.g., “I might be afraid to confide my innermost feelings to(the other person)].” Items are scored on a 5-point scale from 1 (not at all true of me) to 5 (extremely true of me) with higher scores indicating higher fear of engaging in behaviors that demonstrate vulnerability. The Fear of Intimacy Scale shows good reliability and validity in Chinese student samples (53). In the present study, the Cronbach’s alpha coefficient for this scale was 0.871 [95 %CI (0.144, 0.183)].

#### Attachment avoidance

Attachment avoidance was measured by the Chinese revised version (54) of the Experiences in Close Relationships–Relationship Structures Questionnaire (ECR-RSQ; (55)). It is a seven-item self-report instrument that measures attachment dimensions (avoidance and anxiety) in different types of close relationships. The avoidance components in a romantic relationship of the revised version of ECR-RSQ were conducted in this survey, consisting of four items (e.g., “I usually discuss my problems and concerns with this person.”). Each item is scored on a 7-point Likert scale from 1 (strongly disagree) to 7 (strongly agree), with higher scores on each item indicating higher avoidance. Peng (54) illustrated that the revised version of ECR-RSQ shows favorable psychometric characteristics in Chinese college students. In the present study, the Cronbach’s alpha coefficient for this scale was 0.854 [95 %CI (0.555, 0.634)].

#### Subjective wellbeing

Subjective wellbeing was measured by two scales according to its definition (5), with positive/negative affect and life satisfaction, respectively. The positive and negative emotions were measured by The Positive and Negative Affect Scale [PANAS; (56)], which is a 20-item measurement of affect. Ten items evaluate positive affect (e.g., “interested”), and 10 items assess negative affect (e.g., “distressed”). Participants used a five-item Likert scale (from 1 “very slightly or not at all” to 5 “extremely”) to rate their current mood. Higher scores on each sub-scale indicate greater positive or negative affect. In this study, Cronbach’s alpha for positive and negative affects was 0.882 [95 %CI (0.394, 0.463)] and 0.912 [95 %CI (0.457, 0.543)], respectively.

Life satisfaction was measured by the Satisfaction with Life Scale [SWLS; (57)]. It is a five-item self-report measure of life satisfaction (e.g., “In most ways my life is close to ideal”). Respondents are required to rate the extent to which they agree with each item on a 7-point Likert scale (from 1 “strongly disagree” to 7 “strongly agree”). Scores for 5 items are added, with higher scores indicating greater life satisfaction. Cronbach’s alpha for SWLS was 0.855 [95 %CI (0.504, 0.580)] in this study.

Based on previous studies (58, 59), we sum the scores of SWLS and PANAS (positive emotions and negative emotions) to evaluate an individual’s whole subjective wellbeing.

### Data analyses

The descriptive and correlational analyses were conducted in SPSS version 25.0. Mplus version 7.4 was used to analyze the hypothesized multiple mediating pathways and an alternative model, and 95% bias-corrected bootstrap confidence intervals based on 1,000 bootstrap samples were conducted to test the statistical significance of the hypothesized indirect pathways.

## Results

### Descriptive statistics and correlations

Mean values, standard deviations, and bivariate correlations among all the study variables are shown in Table 2. AEE was significantly positively associated with fear of intimacy, while significantly negatively associated with subjective wellbeing. Fear of intimacy was significantly positively associated with attachment avoidance, but significantly negatively associated with subjective wellbeing. Also, attachment avoidance was significantly associated with subjective wellbeing. However, attachment avoidance is not significantly correlated with ambivalence over emotional expression (*p* > 0.05). But, we still conduct a multiple regression model based on our hypothesis, since the bivariate correlations share different aims and functions with regression (60).

**TABLE 2:** Mean values, standard deviations (SD), and correlations among study variables (*N* = 555).

	*M*	SD	1	2	3	4	5	6
1. AEE	5.05	0.862	1					
2. FOI	2.64	0.508	0.176**	1				
3. AA	3.26	1.293	–0.078	0.499**	1			
4. SWB	5.22	2.470	–0.093*	–0.322**	–0.392**	1		
5. Age	–	–	0.041	–0.072	–0.108*	0.080	1	
6. Gender	–	–	–0.127*	–0.002	–0.050	–0.019	–0.131**	1

M, mean; SD, standard deviation; AEE, ambivalence over emotional expression; FOI, fear of intimacy; AA, attachment avoidance; SWB, subjective wellbeing; *p < 0.05, **p < 0.01, ***p < 0.001.

### Mediation analyses

We further analyzed the mediating effects of fear of intimacy and attachment avoidance. We conducted a multiple mediation model using Mplus code, with AEE as the predictor, fear of intimacy as the first mediator, attachment avoidance as the second mediator, subjective wellbeing as the dependent variable, and gender and age as control variables. According to the statistical indices (61), the results suggested that hypothesized multiple mediation model had an acceptable fit (χ^2^/df ratio = 2.810, CFI = 0.975, TLI = 0.924, RMSEA = 0.057, SRMR = 0.030).

**FIGURE 1:**
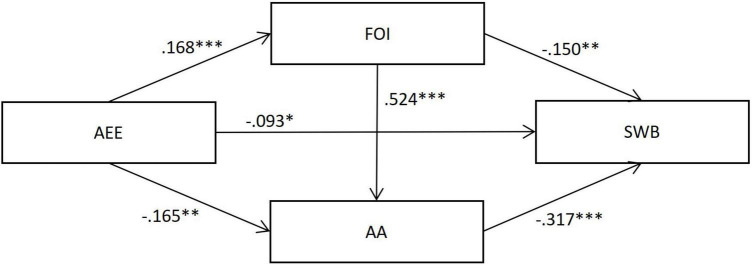
The structural equation model regarding the mediating effects of fear of intimacy and attachment avoidance on the association between ambivalence over emotional expression and subjective wellbeing. Standardized regression coefficients are presented after controlling for age and gender. ^∗∗∗^*p* < 0.001, ^∗∗^*p* < 0.01, ^∗^*p* < 0.05.

Results from the pathway analysis strongly supported our hypotheses. As shown in Figure 1 and Table 3, there were three significant indirect pathways between the association of ambivalence over emotional expression and subjective wellbeing: the specific indirect effects of (a) fear of intimacy, (b) attachment avoidance, and (c) the chain indirect effect of both. Meanwhile, the direct effect of AEE on subjective wellbeing was significant when considering the above indirect effects. Notably, based on Wen et al.’s (62) classification of mediation effect, the pathway analysis of the mediator role of attachment avoidance independently showed a suppression effect, while the direct effect of AEE on subjective wellbeing was negative (β = −0.074) and the indirect effect of attachment avoidance was positive (β = 0.042), which means the negative effect of AEE on subjective wellbeing is suppressed by attachment avoidance.

**TABLE 3:** Results of mediation analyses.

				Bias corrected	
	Estimate	S.E.	Est./S. E.	Lower 2.5%	Upper 2.5%
Total effect	–0.075	0.037	–2.028	–0.150	–0.004
Direct effect	–0.074	0.036	–2.059	–0.144	–0.003
AEE → FOI → SWB	–0.020	0.010	–2.028	–0.046	–0.005
AEE → AA → SWB	0.042	0.016	2.537	0.014	0.079
AEE → FOI → AA → SWB	–0.022	0.008	–2.852	–0.041	–0.011

### Alternative model

**FIGURE 2:**
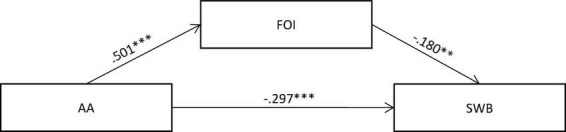
Mediating paths between attachment avoidance and subjective wellbeing *via* fear of intimacy. Standardized regression coefficients are presented after controlling for age and gender. ^∗∗∗^*p* < 0.001, ^∗∗^*p* < 0.01, ^∗^*p* < 0.05.

Finally, we assessed the alternative mediation model, with attachment avoidance as the predictor, fear of intimacy as the mediator, and subjective wellbeing as the dependent variable. The results are shown in Figure 2, revealing that fear of intimacy could act as a partial mediator between the relationship of attachment avoidance and subjective wellbeing (χ^2^/*df* ratio = 1.862, CFI = 0.993, TLI = 0.975, RMSEA = 0.040, SRMR = 0.016), showing an acceptable fit. Specifically, attachment avoidance had a significant direct effect on subjective wellbeing [β = −0.715, 95% CI (-0.958, -0.452)] and a significant indirect effect on subjective wellbeing through fear of intimacy [β -0.218, 95% CI (-0.340, -0.095)], with the total effect of attachment avoidance on subjective wellbeing [β -0.933, 95% CI (-1.127, -0.706)]. These results may point to multidirectional links among the variables studied. In the section “Discussion,” this issue will be further discussed.

## Discussion

The present study focused on a multiple mediation model which examined the relationship between AEE and subjective wellbeing with the mediating roles of fear of intimacy and attachment avoidance among Chinese college students who are involved in a romantic relationship in the context of the COVID-19 outbreak. First, we found that higher levels of AEE could predict a lower level of subjective wellbeing. Those consequences also verified that people with high AEE are likely to deteriorate their subjective wellbeing even in a culture that encourages emotional suppression, and similar results could be found where AEE was negatively associated with pain or stress symptoms (63), which implies that the negative influence of ambivalence over emotional expression might show cross-culture consistency.

### The mediating role of fear of intimacy

Consistent with our hypothesis, the present study illustrated that fear of intimacy had a significant mediating effect between AEE and subjective wellbeing, which means AEE could negatively predict subjective wellbeing *via* the increasing of FOI among college students in love. To be specific, AEE was positively correlated with fear of intimacy and in turn decreased the evaluation of subjective wellbeing. Based on the theory of ambivalence over emotional expression (16), individuals with high AEE usually feel ambivalent when expressing their emotions because they are willing to express their feelings but scared of negative feedback from others. Such feelings are likely to turn into an internal conflict, so in order to mitigate that conflict, they tend to develop emotional suppression as a strategy in daily lives so that they would not be obsessed with the choice of whether or not to express their feelings (18). Also, such an emotional suppression strategy can also be applicable to other relationships, even for romantic relationships that serve as important emotional support for the individual (64), leading to a stronger fear of intimacy.

Furthermore, intimacy is a dynamic relational process involving self-disclosure (44), and Aron et al. (65) emphasized the importance of self-disclosure to increase intimacy in relationship construction, so people with a high fear of intimacy resulting from strong AEE usually have dissatisfaction with romantic relationships. More importantly, such inhibition in romantic relationships triggered by AEE can also expand to a more general aspect, resulting in internalized symptoms such as depression, stress, and social connectedness problems (45), all of which are closely related to a decline in subjective wellbeing.

### The mediating role of attachment avoidance

Although the present study found that the mediating effect of attachment avoidance is significant, it is unexpected that our statistical analysis results illustrated that attachment avoidance played an inhibitory role in the relationship between AEE and subjective wellbeing, suggesting that attachment avoidance could buffer the negative relationship between AEE and subjective wellbeing. This consequence can be explained by the theory of stress (66) and the importance of a romantic relationship (34). Stress occurs when people perceived their environment as problematic (67). Given people with high AEE find difficulties in attaining a sense of social connection *via* emotional expression (17), it is obvious that in the context of COVID-19, they might perceive their situation as highly problematic. But the longing for a social relationship is an eternal pursuit of human beings (68), and their romantic partner could play a pivotal role in times of difficulty or stress (35). Therefore, in order to fulfill their desire to be connected, people who have difficulty expressing their feelings but are in a romantic relationship may turn to their lovers. For example, Ratelle (64) found that a romantic partner was an important source of support, and college students’ wellbeing was the highest when their romantic partner is perceived as highly supportive. What is more, Schacter et al. (69) indicated that high-quality relationships can compensate for rejection from peer groups. Based on these perspectives, it could be assumed that individuals’ romantic relationships might make up for the negative experiences (e.g., stress) from individual normal social interaction, which means high AEE individuals are more likely to attach to their romantic partners rather than avoid them when they find it hard to get along with peers.

More importantly, such a result does not contradict the result of fear of intimacy, which indicated people with high AEE tend to inhibit their emotions in romantic relationships. According to the coping theory (70), there are two types of coping strategies for solving stresses: problem-focus and emotion-focus. The problem-focused approach attempts to alter the source of stress, while the emotion-focused approach aims to handle the emotions in stressful situations. To give an existing finding, people with high AEE still suppress their emotions and feelings in romantic relationships, and they are more likely to take problem-focused approaches, so they might tell negative facts to their partner instead of their feelings about it. However, indeed, the self-disclosure of emotions is a better predictor of relationships’ quality compared with factual expression (71). It might be assumed that although their factual expression with partners may suppress the negative effect of AEE on subjective wellbeing in a short term, it could still impair their romantic relationships, which would eventually lead to a decline in SWB in the future. However, more studies are needed to prove this assumption.

### The sequential mediation model

Findings from the present study revealed that AEE could also influence subjective wellbeing through serial mediation by fear of intimacy and attachment avoidance. Previous research has demonstrated that fear of intimacy is related to attachment difficulties (72). Based on the interpersonal model of intimacy (73), a romantic relationship that involves self-disclosure and responsiveness may feel rewarding. In contrast, those who choose not to express their feelings in romantic relationships were unable to perceive related responses from partners. As a result, they might perceive their partner’s inability to understand their feelings, leading to more avoidant behaviors in relationships. Eventually, such feelings would contribute to dissatisfaction with relationships and even expand to a more general relationship (e.g., friends and classmates) in daily life (40, 47), which could directly deteriorate subjective wellbeing (15). Overall, AEE can exert a direct influence on college students’ subjective wellbeing or indirectly influences subjective wellbeing through enhancing fear of intimacy and attachment avoidance sequentially.

Additionally, given that there are some conflicts about the relationships between fear of intimacy and attachment avoidance, which indicated that the FOI and AA might reciprocally influence each other, so the present study also investigated the mediating role of fear of intimacy in the relationship between attachment avoidance and subjective wellbeing. We found fear of intimacy mediated the influence of attachment avoidance on subjective wellbeing. More specifically, the results of the alternative mediation model confirm the hypothesis that greater attachment avoidance also predicted a higher level of fear of intimacy. Similar results could be seen in previous studies which also demonstrated that attachment avoidance could negatively predict fear of intimacy (74). To sum up, individuals with higher attachment avoidance dismiss the importance of interpersonal relationships and rely solely on themselves (37), leading to emotional distance from romantic relationships.

## Limitations and implications

Because the outbreak of COVID-19 led to numerous psychological issues (1, 2, 4) without enough studies indicating the precaution against that disease, the present study particularly aimed to investigate some factors which might contribute to individuals’ subjective wellbeing and find possible and related intervention based on our results. However, the results of this study should be interpreted in the context of its limitations. First, due to the cross-sectional design adopted in our study, we cannot indicate the causality of the relationships between COVID-19 and psychological factors. Therefore, more experimental and longitudinal studies should be adapted to clarify the direction of the effects of COVID-19 in the future.

Second, although we found the underlying relationships between AEE, attachment avoidance, fear of intimacy, and subjective wellbeing, it is hard to conclude the influence of COVID-19 on that relationship based on our results, so more studies, especially comparison studies, should be applied in the future. Third, because the participants of the present study were employed from three universities in Zhengzhou city and the three universities are located in the same city, some characteristics might be different. Future studies should pay attention to those characteristics. Last but not least, although attachment avoidance played the role of a suppressor in the relationship between AEE and subjective wellbeing, we could not illustrate whether or not that function is valid for a long period, so more longitudinal studies should be adapted. It also raised an interesting question of whether or not the romantic relationship could partly or fully replace the function of peer relationship in some aspects, or compensate for the loss in daily social interactions.

Despite its limitations, the present study still revealed the effects of AEE and the mediating roles of fear of intimacy and attachment avoidance on subjective wellbeing in the context of COVID-19. Due to our research design, we could not draw substantive conclusions about the pandemic influence, but we found that the underlying model could be meaningful when people want to intervene in the mental health of college students. These results provided a more comprehensive conceptualization of how emotional expression conflict is associated with college students’ general evaluation of their lives and were helpful to understand the function of romantic relationships in those relationships. These results point to potential intervention possibilities in promoting college students’ subjective wellbeing and intimacy. In the ecological model of individual development, families and schools are the most basic units of analysis, and their interactions affect the development of youth (75). So, establishing a supportive circumstance that encourages students to express their feelings freely is imperative for their mental health or intimacy development when people are going through the challenges of a pandemic. Considering AEE is rooted in the fear of potential negative feedback in social interaction (16). It is also helpful to apply Rational Emotive Behavior Therapy (REBT) in order to cultivate students’ abilities to prevent over-expectation of negative consequences in social interaction (76). For example, students can be guided to find objective evidence of rejection when getting along with others, and if they cannot find any cues of negative feedback, they may be aware of their cognition bias. Besides, the present study also indicated the function of fear of intimacy and attachment avoidance in the relationship between AEE and subjective wellbeing, and they could reciprocally influence each other, which means intervening in one of them can potentially improve the other. Some treatments could be applied, for example, studies on Functional Analytic Psychotherapy have found that the treatment is effective in reducing fear of intimacy (77). Overall, because of the lockdown policy, individuals’ physical interactions and activities are largely restricted, and hence relevant departments and institutions should pay more attention to alleviating students’ psychological difficulties *via* communication and fostering some protective factors (e.g., perceived social support and mindfulness).

## Conclusion

In conclusion, despite its limitations, the current study contributed to a key point of understanding the multiple mediation model between AEE and the subjective wellbeing of Chinese college students. We found that fear of intimacy and attachment avoidance served as a potential mechanism in the relationship between AEE and subjective wellbeing, with fear of intimacy independently mediating that. Surprisingly, the analysis results showed that attachment avoidance played as a suppressor in such links. More importantly, the current study indicated fear of intimacy and attachment avoidance mediated the link between ambivalence over emotional expression and subjective wellbeing sequentially. We also illustrated that fear of intimacy and attachment avoidance could reciprocally affect each other, which means the fear of intimacy could also mediate the relationship between attachment avoidance and subjective wellbeing. Given that the COVID-19 outbreak seriously damaged college students’ psychological health, our results could be meaningful for related intervention.

## Data availability statement

The original contributions presented in this study are included in the article/supplementary material, further inquiries can be directed to the corresponding author/s.

## Ethics statement

The studies involving human participants were reviewed and approved by Ethical Committee of Zhengzhou University. The patients/participants provided their written informed consent to participate in this study.

## Author contributions

YW and ZZ designed the research and wrote the manuscript. XW performed the research. ZZ designed the structure and performed the calculations. YL reviewed the manuscript and supervised the project. All authors approved the submitted version.

## References

[1] World Health Organization [WHO]. *Coronavirus Disease (COVID-19) Pandemic (Questions and Answers).* Geneva: WHO (2020).

[2] Germani A Buratta L Delvecchio E Mazzeschi C. Emerging adults and COVID-19: the role of individualism-collectivism on perceived risks and psychological maladjustment. *Int J Environ Res Public Health.* (2020) 17:3497. 10.3390/ijerph17103497 32429536 PMC7277425

[3] Banica I Sandre A Shields GS Slavich GM Weinberg A. The error-related negativity (ERN) moderates the association between interpersonal stress and anxiety symptoms six months later. *Int J Psychophysiol.* (2020) 153:27–36. 10.1016/j.ijpsycho.2020.03.006 32277956 PMC7335004

[4] Pigaiani Y Zoccante L Zocca A Arzenton A Menegolli M Fadel S Adolescent lifestyle behaviors, coping strategies and subjective wellbeing during the COVID-19 pandemic: an online student survey. *Healthcare (Basel, Switzerland).* (2020) 8:472. 10.3390/healthcare8040472 33182491 PMC7712064

[5] Diener E. New findings and future directions for subjective well-being research. *Am Psychol.* (2012) 67:590–7. 10.1037/a0029541 23163434

[6] Chida Y Steptoe A. Positive psychological well-being and mortality: a quantitative review of prospective observational studies. *Psychosom Med.* (2008) 70:741–56. 10.1097/PSY.0b013e31818105ba 18725425

[7] Ye B Li L Wang P Wang R Liu M Wang X Social anxiety and subjective well-being among Chinese college students: a moderated mediation model. *Pers Individ Differ.* (2021) 175:110680. 10.1016/j.paid.2021.110680

[8] Wen F Ye H Zuo B Han S Zhu J Ke W The association between insecurity and subjective well-being among youth during the COVID-19 outbreak: a moderated mediation model. *J Affect Disord.* (2022) 297:486–94. 10.1016/j.jad.2021.10.091 34715194 PMC8612099

[9] Armitage R Nellums LB. COVID-19 and the consequences of isolating the elderly. *Lancet Public Health.* (2020) 5:e256. 10.1016/S2468-2667(20)30061-X 32199471 PMC7104160

[10] Zhang P Wang HB. Mediating effect of regulatory emotional self-efficacy on relationships among neuroticism, extraversion and subjective well-being in college students. *Chin Ment Health J.* (2015) 29:139–44. 10.3969/j.issn.1000-6729.2015.02.012

[11] Brockmeyer T Grosse Holtforth M Krieger T Altenstein D Doerig N Friederich H-C Ambivalence over emotional expression in major depression. *Pers Individ Differ.* (2013) 54:862–4. 10.1016/j.paid.2012.12.002

[12] Xie X Guo Q Wang P. Childhood parental neglect and adolescent internet gaming disorder: from the perspective of a distal—proximal—process—outcome model. *Child Youth Serv Rev.* (2021) 120:105564. 10.1016/j.childyouth.2020.105564

[13] Vigl J Strauss H Talamini F Zentner M. Relationship satisfaction in the early stages of the COVID-19 pandemic: a cross-national examination of situational, dispositional, and relationship factors. *PLoS One.* (2022) 17:e0264511. 10.1371/journal.pone.0264511 35239691 PMC8893701

[14] Demirtas SC Tezer E. Romantic relationship satisfaction, commitment to career choices and subjective well-being. *Procedia Soc Behav Sci.* (2012) 46:2542–9. 10.1016/j.sbspro.2012.05.519

[15] Holder MD Love AB Timoney LR. The poor subjective well-being associated with alexithymia is mediated by romantic relationships. *J Happ Stud.* (2015) 16:117–33. 10.1007/s10902-014-9500-0

[16] King LA Emmons RA. Conflict over emotional expression: psychological and physical correlates. *J Pers Soc Psychol.* (1990) 58:864–77. 10.1037/0022-3514.58.5.864 2348373

[17] Bryan JL Lucas S Quist MC Steers M-LN Foster DW Young CM God, can i tell you something? The effect of religious coping on the relationship between anxiety over emotional expression, anxiety, and depressive symptoms. *Psychol Relig Spiritual.* (2016) 8:46–53. 10.1037/rel0000023 27019677 PMC4808057

[18] Lu Q Tsai W Chu Q Xie J. Is expressive suppression harmful for Chinese American breast cancer survivors? *J Psychosom Res.* (2018) 109:51–6. 10.1016/j.jpsychores.2018.03.171 29773152 PMC8054769

[19] Sudo N. The positive and negative effects of the COVID-19 pandemic on subjective well-being and changes in social inequality: evidence from prefectures in Japan. *SSM Popul Health.* (2022) 17:101029. 10.1016/j.ssmph.2022.101029 35079619 PMC8776341

[20] Wang C Wong CCY Lu Q. The pain of ambivalence over emotional expression. *Int J Behav Med.* (2018) 25:216–22. 10.1007/s12529-017-9696-6 29134581

[21] Sue DW Sue D. *Counselling the Culturally Diverse: Theory and Practice.* 5th ed. New York, NY: Wiley (2008).

[22] Figueiredo MI Fries E Ingram KM. The role of disclosure patterns and unsupportive social interactions in the well-being of breast cancer patients. *Psychooncology.* (2004) 13:96–105. 10.1002/pon.717 14872528

[23] Fulmer CA Gelfand MJ Kruglanski AW Kim-Prieto C Diener E Pierro A On “feeling right” in cultural contexts: how person-culture match affects self-esteem and subjective well-being. *Psychol Sci.* (2010) 21:1563–9. 10.1177/0956797610384742 20876880

[24] King LA. Emotional expression, ambivalence over expression, and marital satisfaction. *J Soc Pers Relationsh.* (1993) 10:601–7. 10.1177/0265407593104008

[25] Gomes AP Soares AL Kieling C Rohde LA GonçalvesI H. Mental disorders and suicide risk in emerging adulthood: the 1993 Pelotas birth cohort. *Rev Saude Publica.* (2019) 53:96. 10.11606/s1518-8787.20190530012356 31644774 PMC6802944

[26] Imran S Jackson S. Attachment relationships and psychological distress in young adults: the mediating role of self-esteem. *J Affect Disord Rep.* (2022) 8:100328. 10.1016/j.jadr.2022.100328

[27] Descutner CJ Thelen MH. Development and validation of a fear ofintimacy scale. *Psychol Assess.* (1991) 3:218–25. 10.1037/1040-3590.3.2.218

[28] Mashek DJ Sherman MD. Desiring less closeness with intimate others. In: Mashek DJ Aron AP editors. *Handbook of Closeness and Intimacy.* Mahwah, NJ: Lawrence Erlbaum Associates Publishers (2004). p. 343–56.

[29] Obeid S Sacre H Haddad C Akel M Fares K Zakhour M Factors associated with fear of intimacy among a representative sample of the Lebanese population: the role of depression, social phobia, self-esteem, intimate partner violence, attachment, and maladaptive schemas. *Perspect Psychiatr Care.* (2020) 56:486–94. 10.1111/ppc.12438 31549436

[30] Riggs DS. Traumatized relationships: symptoms of posttraumatic stress disorder, fear of intimacy, and marital adjustment in dual trauma couples. *Psychol Trauma Theory Res Pract Policy.* (2014) 6:201–6. 10.1037/a0036405

[31] Martin AD Mathes BM Schmidt NB. Fear of intimacy and hoarding symptoms: the mediating role of object attachment. *J Obsessive Compuls Relat Disord.* (2022) 32:100702. 10.1016/j.jocrd.2021.100702

[32] Toh SH Kanter JW Keenan ME Berlin KS. Testing functional analytic psychotherapy’s mediational model of change in social connectedness for people with fear of intimacy. *J Context Behav Sci.* (2022) 24:18–22. 10.1016/j.jcbs.2022.02.002

[33] Maitland DW. Experiential avoidance and fear of intimacy: a contextual behavioral account of loneliness and resulting psychopathology symptoms. *J Context Behav Sci.* (2020) 18:193–200. 10.1016/j.jcbs.2020.10.002

[34] Reis HT Collins WA Berscheid E. The relationship context of human behavior and development. *Psychol Bull.* (2000) 126:844–72. 11107879 10.1037/0033-2909.126.6.844

[35] Gleason ME Iida M Bolger N Shrout PE. Daily supportive equity in close relationships. *Pers Soc Psychol Bull.* (2003) 29:1036–45. 10.1177/0146167203253473 15189621

[36] Bartholomew K. Avoidance of intimacy: an attachment perspective. *J Soc Pers Relationsh.* (1990) 7:147–78. 10.1177/0265407590072001

[37] Mikulincer M Shaver PR. *Attachment in Adulthood: Structure, Dynamics, and Change.* New York, NY: Guilford Press (2007).

[38] Joshanloo M. Fear and fragility of happiness as mediators of the relationship between insecure attachment and subjective well-being. *Pers Individ Differ.* (2018) 123:115–8. 10.1016/j.paid.2017.11.016

[39] Kalkotan ZZ. *The Role of Attachment Dimensions, Relationship Status, and Gender in the Components of Subjective Well-Being*. Ph D Thesis. Ankara: Middle East Technical University (2008).

[40] Wickham RE Warren SL Reed DE Matsumoto MK. Attachment and perceived authenticity across relationship domains: a latent variable decomposition of the ECR-RS. *J Res Pers.* (2018) 77:126–32. 10.1016/j.jrp.2018.10.005

[41] Liu J Jackson T. Testing integrated models of relationship satisfaction among married Chinese couples using the actor–partner interdependence model. *J Soc Pers Relationsh.* (2019) 36:1256–77. 10.1177/0265407518757708

[42] Fitzpatrick J Lafontaine M-F. Attachment, trust, and satisfaction in relationships: investigating actor, partner, and mediating effects. *Pers Relationsh.* (2017) 24:640–62. 10.1111/pere.12203

[43] Feeney JA. Attachment, marital interaction, and relationship satisfaction: a diary study. *Pers Relationsh.* (2002) 9:39–55. 10.1111/1475-6811.00003

[44] Reis HT Shaver P. Intimacy as an interpersonal process. In: Duck S Hay DF Hobfoll SE Ickes W Montgomery BM editors. *Handbook Of Personal Relationships: Theory, Research And Interventions.* Hoboken, NJ: John Wiley & Sons (1988). p. 367–89.

[45] Maitland DW Neilson EC. A proposed model for the role of fear of intimacy and social support in behavioral activation: a cross-sectional analysis. *Curr Psychol.* (2021). [Epub ahead of print]. 10.1007/s12144-021-01766-9 PMC10259829 37313352

[46] Candel O-S Turliuc MN. Insecure attachment and relationship satisfaction: a meta-analysis of actor and partner associations. *Pers Individ Differ.* (2019) 147:190–9. 10.1016/j.paid.2019.04.037

[47] Beffel JH Cary KM Nuttall AK Chopik WJ Maas MK. Associations between the broad autism phenotype, adult attachment, and relationship satisfaction among emerging adults. *Pers Individ Differ.* (2021) 168:110409. 10.1016/j.paid.2020.110409

[48] Mayer JD Salovey P. What is emotional intelligence? In: Salovey P Sluyter DJ editors. *Emotional Development And Emotional Intelligence: Educational Implications.* New York, NY: Basic Books (1997). p. 3–31.

[49] Bowlby J. *A Secure Base: Parent–Child Attachments And Healthy Human Development.* New York, NY: Basic Books (1990).

[50] Liu Y Zhai J Chen X. Cognitive neural characteristics of emotion regulation strategies in people with different attachment styles. *Psychol Sci.* (2016) 39:109–15.

[51] Cassidy J. Emotion regulation: influences on attachment relationships. *Monogr Soc Res Child.* (1994) 59:228–49. 10.2307/1166148 7984163

[52] Baorui C Qiandan X Weiwei L Yixuan H Jiaxin C Jiandong F. The effect of ambivalence over emotional expression on psychological symptom: the mediating role of social constraint. *Psychology.* (2020) 8:305–11. 10.16842/j.cnki.issn2095-5588.2020.05.007

[53] Yang Q Shen M. The impact of exposure to domestic violence on fear of intimacy in college students: the chain mediating role of inferiority complex and sense of security. *Chin J Clin Psychol.* (2022) 30:85–8998.

[54] Peng X Luo C Wang Y. The Chinese version of the experiences in close relationships-relationship structures scale (ECR-RS) assesses the validity and reliability for middle school and college students. *Chin Ment Health J.* (2020) 34:957–63.

[55] Fraley RC Heffernan ME Vicary AM Brumbaugh CC. The experiences in close relationships-relationship structures questionnaire: a method for assessing attachment orientations across relationships. *Psychol Assess.* (2011) 23:615–25. 10.1037/a0022898 21443364

[56] Watson D Clark LA Tellegen A. Development and validation of brief measures of positive and negative affect: the PANAS scales. *J Pers Soc Psychol.* (1988) 54:1063–70. 10.1037/0022-3514.54.6.1063 3397865

[57] Diener E Emmons RA Larsen RJ Griffin S. The satisfaction with life scale. *J Pers Assess.* (1985) 49:71–5. 10.1207/s15327752jpa4901_13 16367493

[58] Li C Shi X Dang J. Online communication and subjective well-being in Chinese college students: the mediating role of shyness and social self-efficacy. *Comput Hum Behav.* (2014) 34:89–95. 10.1016/j.chb.2014.01.032

[59] Sheng R Liu T Wang S Yu S Xu W. Mindfulness and late adolescents’ subjective well-being: the serial mediating roles of rejection sensitivity and self-esteem. *Pers Individ Differ.* (2022) 195:111707. 10.1016/j.paid.2022.111707

[60] Shmueli G. To explain or to predict? *Stat Sci.* (2010) 25:289–310. 10.1214/10-STS330

[61] Hu L Bentler PM. Cutoff criteria for fit indexes in covariance structure analysis: conventional criteria versus new alternatives. *Struct Equ Model.* (1999) 6:1–55. 10.1080/10705519909540118

[62] Wen Z Ye B. Analyses of mediating effects: the development of methods and models. *Adv Psychol Sci.* (2014) 22:731. 10.3724/SP.J.1042.2014.00731

[63] Lu Q Yeung N Man J Gallagher MW Chu Q Deen SH. Ambivalence over emotional expression, intrusive thoughts, and posttraumatic stress symptoms among Chinese American breast cancer survivors. *Support Care Cancer.* (2017) 25:3281–7. 10.1007/s00520-017-3744-2 28500541

[64] Ratelle CF Simard K Guay F. University students’ subjective well-being: the role of autonomy support from parents, friends, and the romantic partner. *J Happ Stud.* (2013) 14:893–910. 10.1007/s10902-012-9360-4

[65] Aron A Melinat E Aron EN Vallone RD Bator RJ. The experimental generation of interpersonal closeness: a procedure and some preliminary findings. *Pers Soc Psychol Bull.* (1997) 23:363–77. 10.1177/0146167297234003

[66] Lazarus R Folkman S. *Stress, Appraisal, and Coping.* New York, NY: Springer Publishing Company (1984).

[67] Islam AK Matti Mäntymäki M Laato S Turel O. Adverse consequences of emotional support seeking through social network sites in coping with stress from a global pandemic. *Int J Inf Manag.* (2022) 62:102431. 10.1016/j.ijinfomgt.2021.102431 34642531 PMC8498008

[68] Zou Y Yang X Li J Li Y Wei M. Differences in automatic emotion regulation after social exclusion in individuals with different attachment types. *Pers Individ Differ.* (2022) 185:111296. 10.1016/j.paid.2021.111296

[69] Schacter HL Lessard LM Juvonen J. Peer rejection as a precursor of romantic dysfunction in adolescence: can friendships protect? *J Adolesc.* (2019) 77:70–80. 10.1016/j.adolescence.2019.10.004 31655375 PMC6885552

[70] Folkman S Lazarus RS. An analysis of coping in a middle-aged community sample. *J Health Soc Behav.* (1980) 21:219–39. 7410799

[71] Laurenceau J-P Barrett LF Pietromonaco PR. Intimacy as an interpersonal process: the importance ofself-disclosure, partner disclosure, and perceived partner responsiveness in interpersonal exchanges. *J Pers Soc Psychol.* (1998) 74:1238–51. 10.1037/0022-3514.74.5.1238 9599440

[72] Scigala DK Fabris MA Badenes-Ribera L Zdankiewicz-Scigala E Longobardi C. Alexithymia and self differentiation: the role of fear of intimacy and insecure adult attachment. *Contemp Fam Ther.* (2021) 43:165–76. 10.1007/s10591-021-09567-9

[73] Reis HT Shaver P. Intimacy as an interpersonal process. In: Reis HT editor. *Relationships, Well-Being and Behaviour.* New York, NY: Routledge (2018). p. 113–43.

[74] Read DL Clark GI Rock AJ Coventry WL. Adult attachment and social anxiety: the mediating role of emotion regulation strategies. *PLoS One.* (2018) 13:e0207514. 10.1371/journal.pone.0207514 30592712 PMC6310265

[75] Bronfenbrenner U. *The Ecology of Human Development: Experiments by Nature and Design.* Cambridge, MA: Harvard University Press (1979).

[76] Kim MA Kim J Kim EJ. Effects of rational emotive behavior therapy for senior nursing students on coping strategies and self-efficacy. *Nurse Educ Today.* (2015) 35:456–60. 10.1016/j.nedt.2014.11.013 25475927

[77] Maitland DW Petts RA Knott LE Briggs CA Moore JA Gaynor ST. A randomized controlled trial of functional analytic psychotherapy versus watchful waiting: enhancing social connectedness and reducing anxiety and avoidance. *Behav Anal. Res Pract.* (2016) 16:103–22. 10.1037/bar0000051

